# Epidemiological investigation and risk factor analysis of acute kidney injury in non-intensive care unit hospitalized patients following neurosurgery

**DOI:** 10.3389/fneur.2026.1856616

**Published:** 2026-08-06

**Authors:** Yan Xu, Wen Li, Chunling Dong, Aihua Zhang

**Affiliations:** 1 Department of Nephrology, Xuanwu Hospital Capital Medical University, Beijing, China; 2 Department of Nephrology, Emergency General Hospital, Beijing, China; 3 School of Computer and Cyber Sciences, Communication University of China, Beijing, China

**Keywords:** epidemiology, neurosurgery, non-ICU settings, postoperative acute kidney injury, risk factors

## Abstract

**Background:**

Acute kidney injury (AKI) after neurosurgery is non-negligible. Given brain-kidney crosstalk, postoperative AKI (PO-AKI) can trigger adverse outcomes, but evidence remains limited for neurosurgical patients in non-ICU settings.

**Methods:**

Data from non-ICU inpatients undergoing neurosurgical procedures were collected from Xuanwu Hospital, Capital Medical University. We observed PO-AKI incidence and performed category-weighted multiple regression to identify risk factors.

**Results:**

Data from 9,692 patients were analyzed, yielding a PO-AKI incidence of 3.9%. Sixteen potential risk variables were identified: male sex or age ≥50 years versus female <50 years; intraoperative/immediate postoperative vasopressor use (OR = 4.129, 95% CI: 3.324–5.129); operation duration ≥120 min (OR = 1.797, 95% CI: 1.597–2.021); contrast agent use (OR = 1.912, 95% CI: 1.674–2.184); BMI category, with BMI < 18.5 kg/m^2^ as reference and overweight/obesity associated with reduced PO-AKI risk; preoperative systolic blood pressure (OR = 1.006, 95% CI: 1.003–1.010); perioperative propofol (OR = 2.041, 95% CI: 1.754–2.376), mannitol (OR = 1.766, 95% CI: 1.588–1.963), proton pump inhibitors (OR = 1.668, 95% CI: 1.472–1.892), urapidil (OR = 1.620, 95% CI: 1.442–1.821), and cephalosporin (OR = 1.283, 95% CI: 1.101–1.495); and preoperative urinary protein (OR = 1.255, 95% CI: 1.154–1.366), serum urea (OR = 1.090, 95% CI: 1.058–1.123), serum creatinine (OR = 1.007, 95% CI: 1.004–1.009), alkaline phosphatase (OR = 1.005, 95% CI: 1.004–1.007), and hemoglobin (OR = 0.988, 95% CI: 0.984–0.991). Model discrimination, assessed using the area under the receiver operating characteristic curve, was 0.785.

**Conclusion:**

The incidence of post-neurosurgical AKI was 3.93% among non-ICU patients. Associated factors included vasopressor use, age ≥50 years, surgery duration ≥120 min, contrast agent use, higher systolic blood pressure, anemia, elevated serum creatinine and urea, proteinuria, higher alkaline phosphatase, and medications including propofol, mannitol, proton pump inhibitors, urapidil, and cephalosporin. Propofol association and lower PO-AKI risk across BMI categories versus BMI < 18.5 kg/m^2^ require validation.

## Introduction

Surgical operation is one of the main causes of Acute Kidney Injury (AKI) (1). Previously, we considered the incidence of postoperative acute kidney injury (PO-AKI) after neurosurgery to be relatively low, however, the reality is precisely the opposite. Recent studies indicated that the incidence of PO-AKI following neurosurgery ranged from 1.3 to 17.1% (2–5). Fluctuations in incidence rates are attributable to the heterogeneity of research designs, observed subjects and study endpoint; however, such incidence rates cannot be ignored. Meanwhile, due to the presence of brain-kidney cross-talk (6), PO-AKI can not only increase postoperative mortality and adverse kidney outcomes but also mediate the occurrence or deterioration of various brain injuries through multiple mechanisms (1). A retrospective study involving patients underwent craniotomy confirmed that compared with the non-PO-AKI cohort, the PO-AKI cohort exhibited a worse mortality, along with increased incidences of ventilator dependence and unplanned intubation, as well as greater complication rates and prolonged hospital stays (7). Unfortunately, it has not received adequate attention.

Few studies related to AKI after neurosurgeries have been executed until now. Moreover, the majority of existing studies have primarily focused on patients undergoing major surgeries or those admitted to the Intensive Care Unit (ICU), overlooking a larger cohort of neurosurgical patients who remain in non-ICU settings postoperatively. Up to 20–30% of AKI cases are preventable (8), and patient factors matter more than the surgery itself in PO-AKI development. Understanding PO-AKI risk factors in neurosurgical patients is essential.

## Methods

### Study design and patients

This was a single-center study. All data related to inpatients who remained in non-ICU settings after neurosurgery were collected from the electronic medical record system of Xuanwu Hospital, Capital Medical University. The dataset used encompasses the period from November 2015 to December 2020.

Inclusion criteria: (1) age ≥ 18 years; (2) patients who remained in non-ICU settings after various neurosurgical procedures; (3) availability of complete serum creatinine (Scr) levels within 30 days preoperatively and 7 days postoperatively. Exclusion criteria included: (1) patients with end-stage kidney disease (ESKD) or kidney replacement (9); (2) AKI within 30 days before surgery. Baseline Scr levels were defined as the values measured within one month before surgery (10), with the measurement closest to the operation used when multiple values were available. Postoperative Scr levels were obtained within 7 days after surgery (11). Demographic data, laboratory findings, surgical parameters, and perioperative medication information for the enrolled patients were collected. Body mass index(BMI)stratification followed the criteria established by the World Health Organization (WHO). We used 120 min (120 min) as a cutoff value for operation time in accordance with previous literature (12). Urine protein and urine occult blood were graded based on the local laboratory standards. In addition to pharmaceutical interventions, our analysis focused predominantly on non-surgical and non-intraoperative risk factors.

### Definition of PO-AKI

Postoperative Scr levels were compared with preoperative baseline values to diagnose PO-AKI according to the 2012 Kidney Disease Improving Global Outcomes work group (KDIGO) criteria (13): Scr increase of ≥0.3 mg/dL (≥26.5 μmol/L) within 48 h or a ≥ 1.5-fold increase in the Scr level compared with baseline values—known or presumed—to have occurred within 7 days postoperatively. If the Scr level was lower than the preoperative baseline level within 7 days after surgery, then it would be taken as the baseline (13).

### Statistical analysis

For all statistical analyses, SPSS 26 was employed to compare the baseline data between the AKI and the non-AKI groups, whereas the R 4.5.3 was used for other study components. Missing data were handled using multiple imputation by chained equations (MICE). Continuous variables were expressed as means ± standard deviation (SD) or medians with interquartile ranges (M(Q)), depending on the distribution of the data. Categorical and ordinal data were presented as frequencies and percentages (n (%)). Meanwhile, Wilcoxon rank sum test was employed for comparisons between groups of both ordinal data and continuous variables. The chi-square test was applied for binary variables such as gender. The reference category was defined as the group with a low incidence or level. During the variable screening phase, candidate risk factors were selected based on statistical significance using projection/debiased LASSO regression (incorporating Westfall–Young multiple correction). Subsequently, a multivariate logistic regression (LR) model was constructed using the stably selected variables. Given the class imbalance in the AKI-positive group observed in this study (AKI incidence is low), a class-weighted LR model was further implemented to identify the final risk factors. A *p*-value < 0.05 was considered statistically significant.

### Ethics

This study was approved by the Ethics Committee of Xuanwu Hospital, Capital Medical University ([2022]228). The requirement for written informed consent was waived due to the retrospective nature of this study. The study protocol conformed to the principles of the Declaration of Helsinki.

## Results

### Baseline data analysis

**Figure 1:**
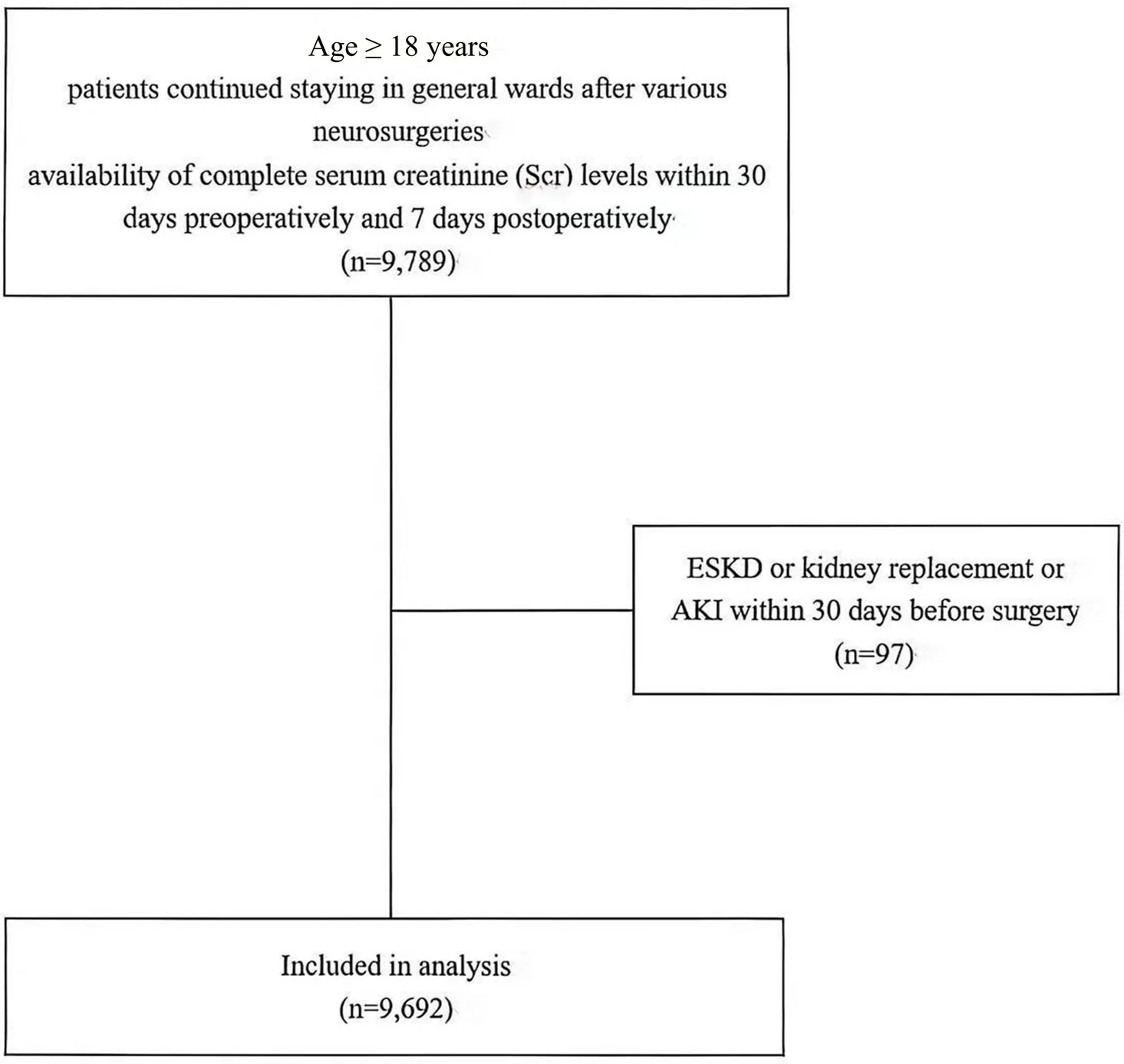
Flow diagram of patient selection.

Data from 9,692 patients who met the inclusion and exclusion criteria were analyzed (Figure 1). There were 5,118 males (52.81%) and 4,574 females (47.19%); the median age was 56 years, ranging from 45 to 65 years. Among the 9,692 non-ICU inpatients who underwent neurosurgery, 381 developed postoperative AKI according to the KDIGO criteria, with an incidence rate of 3.9%.

A comparison of the baseline characteristics including five demographic parameters, seven comorbidities, primary diseases serving as an indication for surgery, preoperative blood pressure, operating duration, contrast agent application, intraoperative blood loss and blood transfusion volume, intraoperative and immediate postoperative vasopressor administration, 26 preoperative lab test items and 12 perioperative medications between PO-AKI and non-AKI groups is presented in Table 1. These results indicated that the incidence of PO-AKI following neurosurgical procedures <120 min (min.) was 3.12%, whereas procedures ≥120 min. Were associated with a PO-AKI incidence of 4.33%. Patients who developed PO-AKI were older and had more comorbidities. Proportion of primary diseases related to surgery differed: cerebrovascular disease was more prevalent in the AKI group. This analysis incorporated intraoperative blood loss, blood transfusion, and vasopressor administration as objective markers of disease severity and perioperative hemodynamic instability. No significant differences in blood loss or transfusion volume were observed between groups; however, vasopressor use was significantly more prevalent in the AKI group. Compared with non-AKI patients, those with AKI had higher preoperative blood pressure and fasting glucose, lower baseline eGFR and higher urea, lower hemoglobin and albumin, higher proteinuria prevalence, and greater exposure to perioperative medications—including contrast agents, propofol, cephalosporins, proton pump inhibitors, mannitol, urapidil, acetaminophen, vancomycin, celecoxib or rofecoxib, ACEIs, and ARBs. Comparison results also showed that the AKI group had a higher ratio of urine erythrocyte or occult blood. Moreover, many indicators related to inflammation or immune status, such as counts of leukocyte, neutrophil, lymphocyte, and neutrophil-to-lymphocyte ratio (NLR), platelet-to-lymphocyte ratio (PLR), were higher than those in the non-AKI group.

**Table 1:** Comparison of baseline data between patients with PO-AKI and without AKI.

Factors	Total (*n* = 9,692)	Non-AKI (*n* = 9,311) M(Q)/n (%)	AKI (*n* = 381) M(Q)/n (%)	χ^2^/Z	*P*
Sex				3.098	0.078
Male	5,118	4,900(52.6)	218(57.2)		
Female	4,574	4,411(47.4)	163(42.8)		
Age (years)		56(19)	59(18)	4.734	<0.001
BMI (kg/m^2^)^1^				0.576	0.564
<18.5	282	263(3.1)	19(5.4)		
18.5 ≤ BMI < 24.0	3,526	3,388(39.4)	138(39.0)		
24.0 ≤ BMI < 28.0	3,589	3,458(40.2)	131(37.0)		
≥28.0	1,551	1,485(17.3)	66(18.6)		
Smoking history				0.030	0.985
N^2^	7,937	7,624(90.2)	313(90.5)		
Y^2^	863	830(9.8)	33(9.5)		
Drinking history				0.424	0.809
N	8,257	7,929(92.5)	328(93.4)		
Y	664	641(7.5)	23(6.6)		
Comorbidity history					
Diabetes/glucose abnormality				26.403	<0.001
N	8,223 (84.8)	7,935 (85.2)	288 (75.6)		
Y	1,469 (15.2)	1,376 (14.8)	93 (24.4)		
Hypertension				32.849	<0.001
N	6,151 (63.5)	5,962 (64.0)	189 (49.6)		
Y	3,541 (36.5)	3,349 (36.0)	192 (50.4)		
Cardiovascular disease				18.017	<0.001
N	8,791 (90.7)	8,469 (91.0)	322 (84.5)		
Y	901 (9.3)	842 (9.0)	59 (15.5)		
Liver disease				122.982	<0.001
N	9,280 (95.7)	8,958 (96.2)	322 (84.5)		
Y	412 (4.3)	353 (3.8)	59 (15.5)		
Heart failure				116.055	<0.001
N	9,635 (99.4)	9,272 (99.6)	363 (95.3)		
Y	57 (0.6)	39 (0.4)	18 (4.7)		
Hyperlipidemia				1.007	0.317
N	8,862 (91.4)	8,519 (91.5)	343 (90.0)		
Y	830 (8.6)	792 (8.5)	38 (10.0)		
Hyperuricemia/gout				13.934	<0.001
N	9,607 (99.1)	9,236 (99.2)	371 (97.4)		
Y	85 (0.9)	75 (0.8)	10 (2.6)		
Primary disease				55.658	<0.001
Cerebrovascular disease	3,718 (38.4)	3,516 (37.8)	202 (53.0)		
Tumor/space-occupying lesion	3,041 (31.4)	2,926 (31.4)	115 (30.2)		
Spine/spinal cord disease	1817 (18.7)	1780 (19.1)	37 (9.7)		
Functional neurosurgery disease	567 (5.9)	562 (6.0)	5 (1.3)		
Trauma/hematoma	244 (2.5)	231 (2.5)	13 (3.4)		
Others	305 (3.1)	296 (3.2)	9 (2.4)		
Preoperative blood pressure					
Systolic pressure	9,156	126(20)	130(24)	4.592	<0.001
Diastolic pressure	9,156	78(12)	80(15)	2.647	0.008
Operating duration (min)				8.216	0.004
<120	3,173	3,074(33.0)	99(26.0)		
≥120	6,519	6,237(67.0)	282(74.0)		
Blood loss (mL)	9,681	10(77.5)	10(75)	0.708	0.479
Blood Transfusion volume (mL)	9,688	0(0)	0(0)	0.018	0.985
Vasopressor use				528.353	<0.001
N	9,344	9,059(97.3)	285(74.8)		
Y	348	252(2.7)	96(25.2)		
Contrast agent				22.012	<0.001
N	7,455	7,198(77.3)	257(67.5)		
Y	2,237	2,113(22.7)	124(32.5)		
Preoperative lab test					
Serum creatinine (μmol/L)		58(19)	57(27)	0.728	0.467
eGFR(ml/min/1.73 m^2^)^3^		107.9(20.9)	105.9(26.5)	3.369	<0.001
Urea (mmol/L)		4.87(1.91)	5.28(2.81)	4.974	<0.001
Albumin (g/L)		39.86(4.66)	39.41(6.11)	3.755	<0.001
Uric acid(μmol/L)		311(118)	305(154)	0.718	0.473
Fasting blood glucose (mmol/L)		4.88(1.16)	5.22(2.27)	7.462	<0.001
Triglyceride (mmol/L)		1.28(0.91)	1.36(0.96)	0.689	0.491
TyG^4^		0.25(0.18)	0.22(0.19)	3.463	<0.001
Cholesterol (mmol/L)		4.15(1.35)	4.11(1.36)	1.451	0.147
LDL-C (mmol/L)^5^		2.52(1.15)	2.49(1.22)	0.896	0.370
HDL-C (mmol/L)^6^		1.13(0.42)	1.07(0.44)	2.475	0.013
Potassium (mmol/L)		3.99(0.43)	4.00(0.46)	0.048	0.962
Sodium (mmol/L)		141(3.0)	140(4.0)	2.546	0.011
Chloride (mmol/L)		104(4)	104(5)	1.426	0.154
Calcium (mmol/L)		2.25(0.15)	2.23(0.17)	2.932	0.003
phosphorus (mmol/L)		1.14(0.23)	1.10(0.28)	3.066	0.002
Hemoglobin (g/L)		136(21)	133(25)	4.336	<0.001
Leukocyte count (10^9^/L)		5.99(2.23)	6.49(3.32)	6.359	<0.001
Platelet count (10^9^/L)		228(78)	222(79)	1.755	0.079
Neutrophil count (10^9^/L)		3.48(1.78)	4.15(2.80)	8.496	<0.001
Lymphocyte count (10^9^/L)		1.84(0.79)	1.635(0.87)	6.888	<0.001
NLR^7^		1.87(1.24)	2.43(2.42)	10.585	<0.001
PLR^8^		124.38(59.69)	140.04(88.17)	5.267	<0.001
Fibrinogen (g/L)		3.13(0.97)	3.34(1.31)	4.727	<0.001
Urine protein^9^				6.509	<0.001
−	7,323	7,098(81.4)	225(68.8)		
±	1,245	1,199(13.8)	46(14.1)		
+	398	363(4.2)	35(10.7)		
2+	59	43(0.5)	16(4.9)		
≥3+	18	13(0.1)	5(1.5)		
urine occult blood^9^				5.088	<0.001
−	7,172	6,946(79.7)	226(69.1)		
±	880	846(9.7)	34(10.4)		
+	604	570(6.5)	34(10.4)		
2+	289	264(3.0)	25(7.6)		
≥3+	98	90(1.0)	8(2.4)		
Perioperative medication					
Propofol				337.140	<0.001
N	8,751	8,511(91.4)	240(63.0)		
Y	941	800(8.6)	141(37.0)		
Cephalosporin				13.285	<0.001
N	2,076	2,023(21.7)	53(13.9)		
Y	7,616	7,288(78.3)	328(86.1)		
Proton pump inhibitor				37.495	<0.001
N	3,294	3,222(34.6)	74(19.4)		
Y	6,398	6,091(65.4)	307(80.6)		
Mannitol				98.804	<0.001
N	7,000	6,810(73.1)	190(49.9)		
Y	2,692	2,501(26.9)	191(50.1)		
Urapidil				103.459	<0.001
N	7,549	7,333(78.8)	216(56.7)		
Y	2,143	1,978(21.2)	165(43.3)		
Acetaminophen				111.421	<0.001
N	7,680	7,460(80.1)	220(57.7)		
Y	2,012	1,851(19.9)	161(42.3)		
Vancomycin				66.547	<0.001
N	8,871	8,566(92.0)	305(80.1)		
Y	821	745(8.0)	76(19.9)		
Celecoxib/Rofecoxib				7.083	0.008
N	8,355	8,009(86.0)	346(90.8)		
Y	1,337	1,302(14.0)	35(9.2)		
Oxycodone				5.661	0.017
N	8,035	7,702(82.7)	333(87.4)		
Y	1,657	1,609(17.3)	48(12.6)		
Somedon				0.471	0.492
N	8,990	8,640(92.8)	350(91.9)		
Y	702	671(7.2)	31(8.1)		
ACEI^10^				196.776	<0.001
N	7,594	7,406(79.5)	188(49.3)		
Y	2,098	1,905(20.5)	193(50.7)		
ARB^11^				10.745	0.001
N	8,616	8,297(89.1)	319(83.7)		
Y	1,076	1,014(10.9)	62(16.3)		

1. BMI: body mass index. 2. N = not; Y = yes. 3. eGFR: estimated Glomerular Filtration Rate based on serum creatinine. 4. TyG: Triglyceride-Glucose index, the formula is that Ln [fasting triglyceride (mg/dl) × fasting blood-glucose (mg/dl)/2]. 5. LDL-C: Low-Density Lipoprotein Cholesterol. 6. HDL-C: High-Density Lipoprotein Cholesterol. 7. NLR: blood Neutrophil-to-Lymphocyte count Ratio. 8. PLR: blood Platelet-Lymphocyte Ratio. 9. Proteinuria and urine occult blood: − = Negative; ± = Trace; + ~ ≥3 + = Urine protein + ~ 4+. 10. ACEI: Angiotensin Converting Enzyme Inhibitor. 11. ARB: Angiotensin Receptor Blockers.

### Subgroup analysis of the incidence of AKI after neurosurgery in different age-sex group

Subgroup analysis was conducted by dividing all the observed subjects into four groups: females <50 years old, males <50 years old, females ≥50 years old, and males ≥50 years old. The differences in the incidence of PO-AKI among these groups were analyzed to determine if they were statistically significant. The outcomes revealed that for both males and females, the incidence of PO-AKI was significantly higher in patients aged ≥50 years compared to those aged <50 years. However, within the ≥50 and <50 age groups, while the incidence of PO-AKI was consistently higher in males than in females, no statistically significant difference was observed in either group. The incidence of PO-AKI in different groups was as follows: 2.6% for females <50 years, 3.0% for males < 50 years, 4.1% for females ≥50 years, and 4.9% for males ≥50 years. Among all patients with PO-AKI, the proportions in the four subgroups were as follows: females <50 years accounted for 10.8%, males <50 years for 13.6%, females ≥50 years for 32%, and males ≥50 years for 43.6% (Tables 2, 3). Compared with females under 50 years (reference, OR = 1), males aged ≥50 years (OR = 1.613, 95% CI 1.347–1.933) and females aged ≥50 years (OR = 1.356, 95% CI 1.153–1.596) had higher odds of PO-AKI, whereas the estimate for males aged <50 years was not statistically significant (OR = 1.113, 95% CI 0.913–1.357; *p* = 0.288) (Table 4).

**Table 2:** Subgroup analysis—analysis of the incidence of AKI after neurosurgery in combination with gender and age.

Sex	Age	*N*	Non-AKI *n* (%)	AKI *n* (%)	/Z	*P*	Proportion (%)^1^
Male	<50	1,707	1,655(33.8)	52(23.9)	9.244	0.002	13.6
	≥50	3,411	3,245(66.2)	166(76.1)			43.6
Female	<50	1,580	1,539(34.9)	41(25.2)	6.591	0.010	10.8
	≥50	2,994	2,872(65.1)	122(74.8)			32.0

1. Proportion: Proportional Distribution of AKI Cases Across Different Subgroups in the Overall AKI Population. The outcomes revealed that for both males and females, the incidence of PO-AKI was significantly higher in patients aged ≥ 50 years compared to those aged < 50 years. The AKI incidence of group female ≥50 was even significantly higher than it in group male <50 years.

**Table 3:** Subgroup analysis—Analysis of the incidence of AKI after neurosurgery in combination with gender and age.

Age	Sex	*N*	Non-AKI n (%)	AKI *n* (%)	/Z	P	Proportion (%)^1^
<50	Female	1,580	1,539(48.2)	41(44.1)	0.608	0.436	10.8
	Male	1,707	1,655(51.8)	52(55.9)			13.6
≥50	Female	2,994	2,872(47.0)	122(42.4)	0.131	0.071	32.0
	Male	3,411	3,245(53.0)	166(57.6)			43.6

1. Proportion: Proportional Distribution of AKI Cases Across Different Subgroups in the Overall AKI Population. Within the ≥ 50 and < 50 age groups, while the incidence of PO-AKI was consistently higher in males than in females, no statistically significant difference was observed in either group.

**Table 4:** Category-weighted multiple LR analysis of risk factors for postoperative AKI in inpatients who remained in non-ICU settings after neurosurgery.

Factors	Regression coefficient	Wald chi-square	*P*	OR	95% CI for OR
Sex / age					
Female <50 years	Reference				
Male <50	0.107	1.129	0.288	1.113	0.913–1.357
Female ≥50	0.305	13.531	<0.001	1.356	1.153–1.596
Male ≥50	0.478	26.991	<0.001	1.613	1.347–1.933
Surgery duration					
<120 min	Reference				
≥120 min	0.586	95.27	<0.001	1.797	1.597–2.021
Contrast agent					
N^1^	Reference				
Y	0.648	91.045	<0.001	1.912	1.674–2.184
BMI^2^					
<18.5 kg/m^2^	Reference				
18.5–<24.0	−0.823	34.593	<0.001	0.439	0.333–0.579
24.0–<28.0	−0.911	40.021	<0.001	0.402	0.302–0.534
≥28.0	−0.901	35.35	<0.001	0.406	0.301–0.548
Systolic blood pressure	0.006	14.213	<0.001	1.006	1.003–1.01
Vasopressor use					
N	Reference				
Y	1.418	164.375	<0.001	4.129	3.324–5.129
Propofol					
N	Reference				
Y	0.713	84.842	<0.001	2.041	1.754–2.376
Mannitol					
N	Reference				
Y	0.568	110.869	<0.001	1.766	1.588–1.963
Proton pump inhibitor					
N	Reference				
Y	0.512	63.888	<0.001	1.668	1.472–1.892
Urapidil					
N	Reference				
Y	0.483	65.906	<0.001	1.62	1.442–1.821
Cephalosporin					
N	Reference				
Y	0.249	10.212	0.0014	1.283	1.101–1.495
Hemoglobin (g/L)	−0.013	50.453	<0.001	0.988	0.984–0.991
Urine protein	0.227	28.189	<0.001	1.255	1.154–1.366
Urea (mmol/L)	0.086	32.168	<0.001	1.09	1.058–1.123
creatinine (μmol/L)	0.007	21.003	<0.001	1.007	1.004–1.009
ALP^3^(U/L)	0.005	33.189	<0.001	1.005	1.004–1.007

1. N = not; Y = yes. 2. BMI: body mass index. 3. ALP: Alkaline phosphatase.

### Category-weighted multiple LR analysis of risk factors for postoperative AKI in inpatients who stayed in non-ICU settings after neurosurgeries

**Figure 2:**
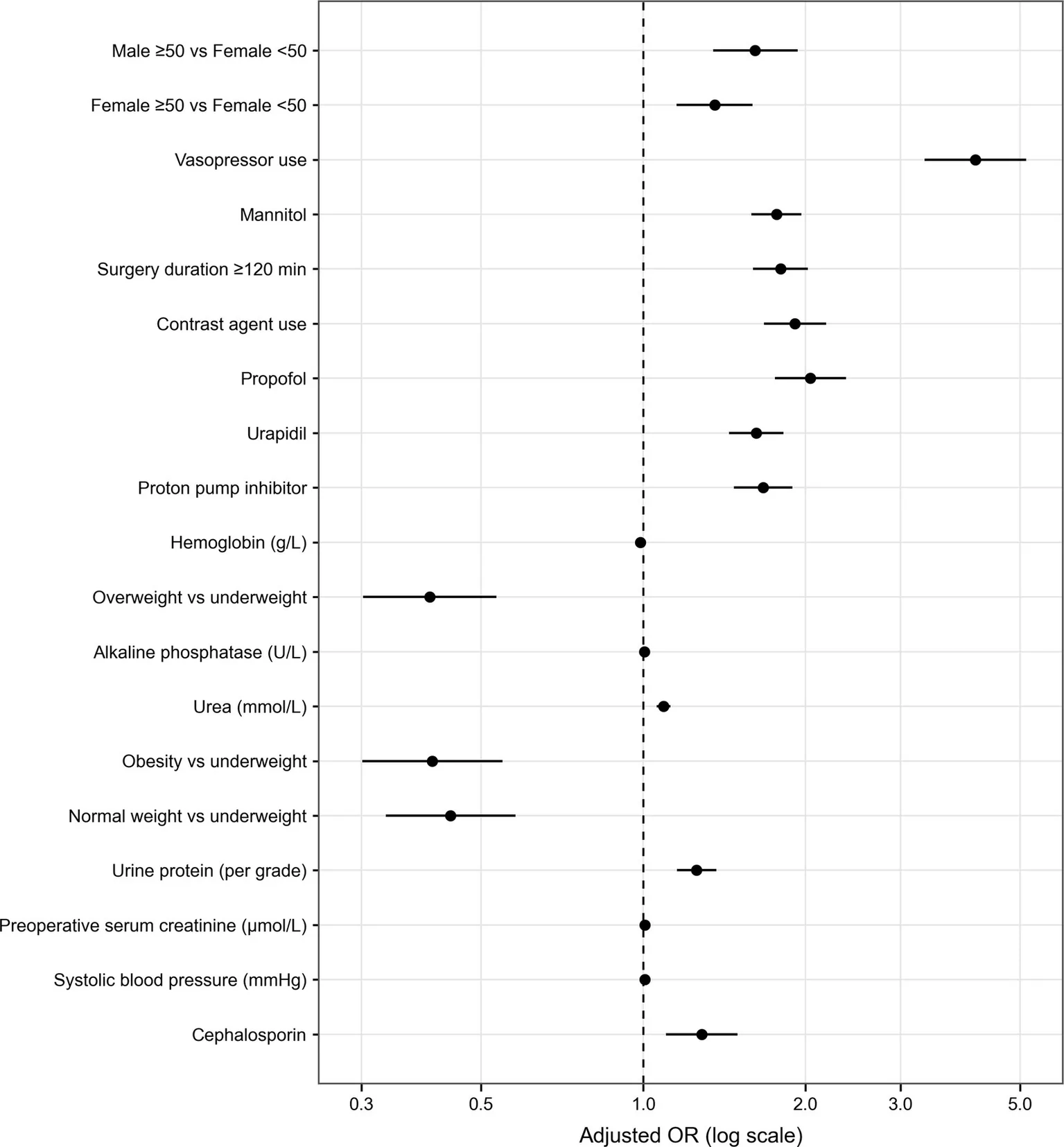
Forest plot of all independent risk factors for acute kidney injury after neurosurgical operations. The dashed line denotes an adjusted odds ratio of 1.0.

**Figure 3:**
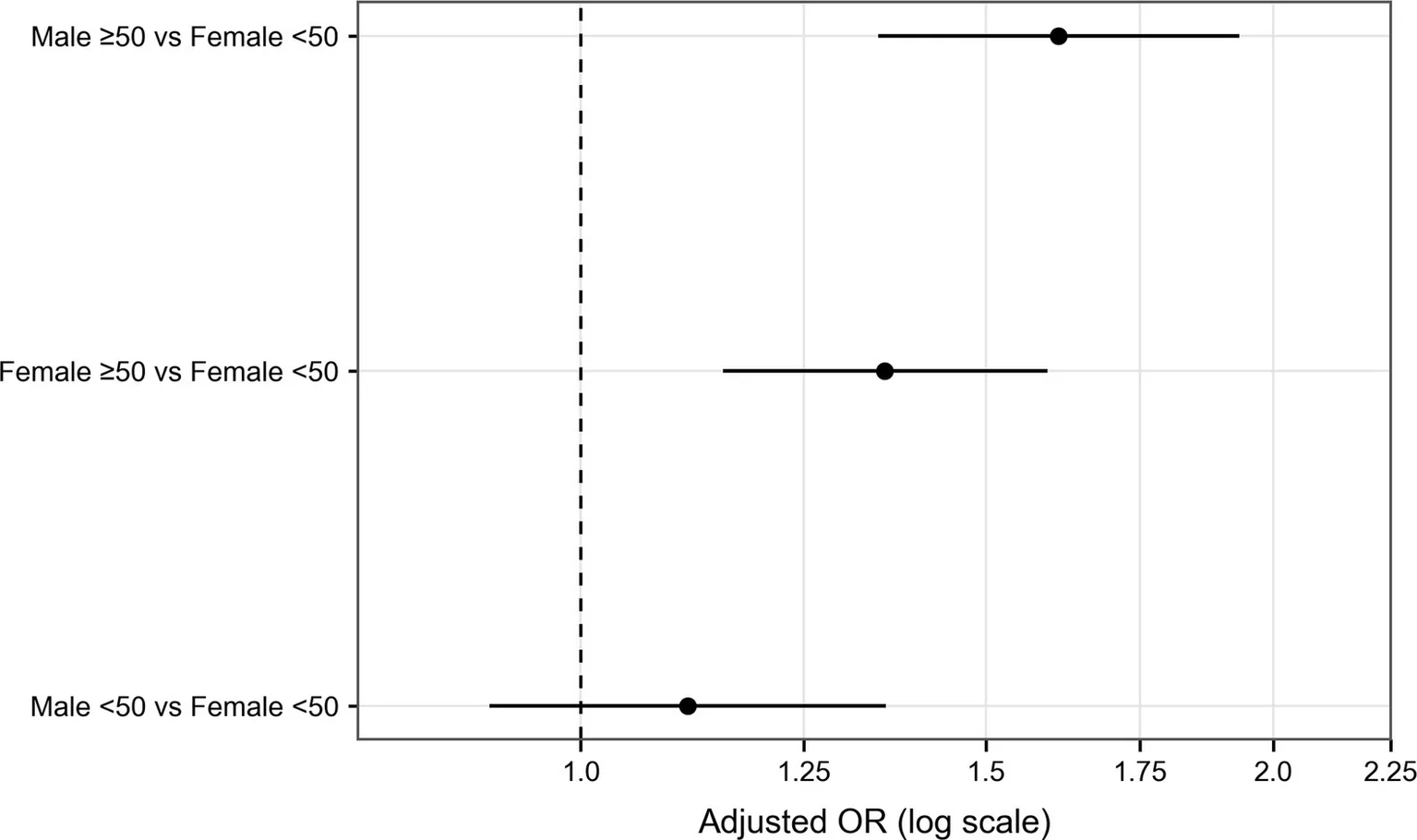
Forest plot for age-sex subgroups. All three other groups were compared to the group Female <50 years old. The dashed line denotes an adjusted odds ratio of 1.0.

We identified candidate variables through stabilized selection with debiased LASSO regression. Subsequently, to account for class imbalance in the outcome, we constructed class-weighted multivariable LR models on each imputed dataset and combined the effect estimates using Rubin’s rules. Ultimately, the category-weighted multiple LR model analysis identified a total of 16 risk factors for AKI in general neurosurgical ward inpatients after surgery (Table 4). Details were as follows: (1) Vasopressor use had the largest effect estimate (OR = 4.129, 95% CI 3.324–5.129). (2) Compared with the female cohort under 50 years old, males and females aged 50 years or older exhibit an increased risk of PO-AKI. (3) Other positively associated perioperative factors included surgery lasting ≥120 min (12), contrast exposure, propofol, mannitol, proton pump inhibitors, urapidil, and cephalosporins. (4) Higher urine protein grade, preoperative serum urea and creatinine, systolic blood pressure, and alkaline phosphatase were positively associated with PO-AKI, whereas hemoglobin was inversely associated. (5) Relative to BMI < 18.5 kg/m^2^, normal weight, overweight, and obesity were associated with lower odds of PO-AKI. The discriminative ability of the model was assessed using the area under the receiver operating characteristic curve (AUROC), which was 0.785 (Table 4 and Figures 2, 3). (5) Multiple perioperative medication exposures demonstrated independent associations with PO-AKI following neurosurgery: propofol, proton pump inhibitors, mannitol, urapidil, and cephalosporin (Table 4 and Figure 2).

## Discussion

Current research on the association between neurosurgical procedures and postoperative AKI remains limited (7), particularly regarding the incidence and risk factors of PO-AKI in hospitalized patients undergoing neurosurgery outside ICU settings. Nevertheless, neurosurgery is a significant precipitating factor for AKI. This study found that even non-major surgeries lasting less than 120 min were associated with a 3.12% incidence of PO-AKI; when surgery duration was ≥120 min, the incidence increased to 4.33%.

AKI not only increases postoperative mortality and risk of chronic kidney disease (CKD), but also contributes to short-term complications including fluid overload, arrhythmia, infection, and hemorrhage, as well as adverse drug reactions (12). Moreover, AKI can disrupt cerebral homeostasis by altering neurotransmitter levels, circulating cytokines, acid–base balance, hemostasis, and drug metabolism. Efferent signals from the central nervous system may increase renal sympathetic activity, promoting renin release and reducing renal blood flow. In turn, brain-kidney interactions in AKI may involve cytokine-mediated damage, oxidative stress, leukocyte extravasation, and impaired cerebral aquaporin function. AKI-related pro-inflammatory and metabolic disturbances can compromise the blood–brain barrier, leading to cerebral edema, inflammation, hemorrhage, and higher mortality (14, 15). Given the bidirectional interplay between the brain and kidneys in maintaining homeostasis, postoperative AKI after neurosurgery warrants close attention and early preventive strategies—even in procedures lasting less than 120 min—to improve patient outcomes.

Our subgroup analysis revealed that AKI prevalence was significantly higher in males aged 50 years and above compared to younger males, with a similar trend observed in females. Although PO-AKI incidence was higher in males than in females across all age groups, these differences were not statistically significant. These findings suggest that age, rather than gender, may be the primary driver of AKI development. PO-AKI incidence was lowest in females under 50 years and highest in males aged 50 years and above. In females aged ≥50 years, incidence exceeded that in males under 50. This pattern indicates that advancing age or the loss of premenopausal protective effects may be key contributors to AKI risk for females. A retrospective study by Privratsky et al. (16) including 390,382 adult inpatients undergoing major non-cardiac, non-renal surgery further supports this: after adjusting for established AKI risk factors, the risk of AKI remained significantly elevated in females over 50 (OR = 1.51, 95% CI 1.43–1.59), males under 50 (OR = 1.90, 95% CI 1.79–2.01), and males over 50 (OR = 2.06, 95% CI 1.96–2.17), relative to females under 50 (reference OR = 1). The difference between our results and those of Privratsky et al. lies in the higher odds ratio observed in females over 50 years of age compared to males under 50, which warrants further investigation to determine whether it stems from differences in study populations or study design. Notably, our study is the first to confirm these patterns specifically in neurosurgical patients with PO-AKI, highlighting the importance of investigating the interplay between female sex, aging, and peri- or postmenopausal age, and hormonal changes may be involved in AKI pathogenesis.

Given the inherent constraints of retrospective study design, we evaluated three intraoperative variables—namely, intraoperative blood loss, red blood cell transfusion volume, and vasopressor administration during surgery and in the immediate postoperative period—as potential contributors to postoperative acute kidney injury (PO-AKI). Among these, vasopressor administration demonstrated the strongest statistically significant association with PO-AKI. Vasopressor use serves as a clinical proxy for perioperative hemodynamic instability—a well-established pathophysiological mechanism underlying PO-AKI—and may also reflect renal vasoconstrictive effects of vasoactive agents (17, 18). In contrast, intraoperative blood loss and red blood cell transfusion exhibited minimal variability across cases: over 80% of procedures were associated with blood loss of 0–10 mL and no red blood cell transfusion, likely limiting statistical power to detect meaningful associations with PO-AKI in this cohort.

Moreover, our study assessed the link between common perioperative medications in neurosurgery and PO-AKI. Weighted multivariable logistic regression showed that propofol, mannitol, proton pump inhibitors, urapidil, and cephalosporin were associated with higher PO-AKI risk. Acetaminophen and vancomycin differed in univariable baseline comparisons but were not retained in the final weighted model. This study identified five commonly used perioperative neurosurgical medications as risk factors for PO-AKI. Nephrotoxic drugs can trigger AKI via direct tubular injury, intratubular obstruction, microvascular changes, or interstitial inflammation. Propofol is widely used in neurosurgery and ICU. The Chinese Clinical Practice Guideline for AKI (2023) (10) recommends it for short-term sedation due to its potential to reduce AKI risk. A study showed no difference in PO-AKI rates between propofol and sevoflurane, but propofol was linked to lower AKD incidence (16.9% vs. 6.3%) (19). Other studies also lack clear evidence linking propofol to kidney damage (1). However, our study was the first to find that propofol may be associated with an increased risk of PO-AKI in neurosurgical patients. Rare mechanistic studies found that propofol impairs renal metabolism. It disrupts oxidative phosphorylation, especially in the outer medulla, promoting a shift to anaerobic glycolysis (20). In mice, propofol preconditioning worsens outer medullary tubular injury after ischemia–reperfusion. Previous publications showed that, in kidney transplant patients, higher intraoperative propofol levels are linked to lower oxidative metabolism, more tubular injury, and poorer long-term kidney outcomes (20). These findings support further clinical study of propofol’s association with PO-AKI.

The association between perioperative cephalosporin use and postoperative acute kidney injury (PO-AKI) remains unclear: some studies have found no link to postoperative acute kidney injury (21, 22), while cephalosporins can indeed cause drug-induced acute kidney injury. Mannitol is widely used in neurosurgery but may increase the risk of PO-AKI, consistent with our study findings. The mechanism may involve elevated serum osmolality and appears dose-dependent (23). In patients having elective brain tumor surgery, preoperative mannitol use carries a higher PO-AKI risk than intraoperative or postoperative use, likely due to faster infusion rates (24). In this study, urapidil was associated with postoperative AKI. It remains unclear whether this reflects direct nephrotoxicity or uncontrolled hypertension causing renal injury. Since elevated preoperative systolic blood pressure correlates with higher PO-AKI risk, the latter is more likely—highlighting the importance of perioperative blood pressure management. Proton pump inhibitors are also widely used and may be nephrotoxic, thus, assess their need before prescribing instead of using them routinely. The association between contrast agents and PO-AKI remains debated. Some studies showed no increased risk with iodinated contrast (25), while others reported a significant association (26). Our study found that using contrast during neurosurgical procedures was an independent risk factor for AKI. The American College of Radiology introduced two terms: contrast-induced AKI, where contrast directly causes injury, and contrast-associated AKI, which includes all AKI within 48 h after contrast use. Although the incidence of contrast-induced AKI is significantly lower than that of contrast-associated AKI, the actual risk remains undetermined (25). Subclinical contrast-associated AKI is increasingly recognized and appears more frequent than clinical AKI after neurosurgery (27, 28). Combining the results of previous and this study, for patients with existing AKI risks, contrast dose, route, and perioperative hydration should be carefully considered.

This study found that, BMI < 18.5 kg/m^2^ increased PO-AKI risk, On the other hand, higher BMI (normal, overweight, or obesity) categories were protective against PO-AKI after neurosurgery, this was similar to the obesity/BMI paradox described in Bellamkonda’s 2024 review (29). For illustration, in people without cardiovascular disease, BMI and mortality follow a J-shaped curve with optimal range 20–25 kg/m^2^; in those with cardiovascular disease, the relationship is U-shaped, with optimal BMI shifting to 25–30 kg/m^2^. A similar BMI paradox may exist in neurosurgical patients or under surgical stress, requiring further study. Some other researchers also confirmed that BMI < 18.5 kg/m^2^ would lead to adverse outcomes; for example, underweight patients with cancer undergoing immunotherapy or peripheral vascular interventions had worse prognoses than those with normal or higher BMI (30, 31). All of these findings indicated that BMI < 18.5 kg/m^2^ may be related to malnutrition, which weakens the ability to withstand stressors such as surgery or cancer. In addition, some of our findings were consistent with prior studies (1, 7): aging is an independent risk factor for post-neurosurgical AKI, with older patients more vulnerable to surgery and worse outcomes after PO-AKI; preoperative kidney dysfunction, especially CKD, increases AKI risk; Anemia is also an independent risk factor, and its severity correlates positively with PO-AKI risk. This current research indicated that major surgeries (>120 min) increase PO-AKI incidence, matching results from the UK-based ISOS study (32). Albuminuria, a marker of glomerular and endothelial injury, is independently linked to PO-AKI (33)—our data confirm this association. Additionally, higher ALP remained independently associated with PO-AKI, but the clinical meaning and mechanism require validation.

However, our study still had certain limitations, including: (1) Due to the single-center nature of the study, multi-center data analysis is needed; (2) The research results are limited by retrospective data, however, the clinical confounding factors of PO-AKI are numerous and interrelated, requiring further prospective studies for validation; (3) Propofol as an independent risk factor for AKI after neurosurgery needs to be verified by multi-center prospective studies.

## Conclusion

This study is the first to analyze data from 9,692 neurosurgical patients admitted to non-ICU settings, with a PO-AKI incidence of 3.93%. Those risk factors included vasopressor use age ≥50 years, surgery duration ≥120 min, contrast agent use, higher systolic blood pressure; preoperative anemia, elevated serum creatinine and urea levels, preoperative proteinuria, higher alkaline phosphatase; and medications such as propofol, mannitol, proton pump inhibitors, urapidil, and cephalosporin. Two novel factors warrant further validation: (1) the association of propofol with PO-AKI, (2) a lower PO-AKI risk in all BMI categories compared to that in the BMI < 18.5 kg/m^2^ category.

## Data Availability

The data presented in this study are available from the corresponding author upon reasonable request. The data are not publicly available due to privacy concerns regarding participant information.
